# Annotations

**Published:** 1898-01-08

**Authors:** 


### ANNOTATIONS.

### Theatres, Churches, and Consumption.

DR. RANSOME has classed consumption in the unpleasant category of "filth diseases." There can be but little doubt that he is right, although the filth is not by any means of necessity a matter of personal uncleanliness. He puts the matter somewhat in this way. There are certain diseases which, although infectious, do not "catch" directly from person to person. The specific organisms which are the causes of these maladies are capable of life even for long periods outside the body, they not only can live but they can grow on organic matter, and on such organic matter they are carried from case to case. Their spread is in fact the consequence of men swallowing or breathing each other's excreta. "What," says Dr. Ransome, "can be conceived as more fitted to come within the designation of filth than the loathsome excretions from the lungs of phthisical patients?" Yet, as is well known, it is the breathing of the dust arising from dried and ground up sputa so deposited that is one great cause of consumption. Dr. Ransome lays especial stress upon the risks we run in churches, concert halls, and theatres. In regard to theatres he says, "The halls and passages and galleries of these places, and even their auditoria, are seldom, if ever, visited by the sunlight. . . . They are imperfectly ventilated. . . . They are true hotbeds and forcing grounds in which may be kept alive, and even cultivated, the germs of tuberculosis and other diseases." But the churches seem to be even worse. "Let anyone gifted with an ordinary sense of smell, or who knows what fresh air means enter almost any of these assemblies for public worship, even after only a short service, and let him describe the atmosphere he will meet on entering. It is charged with 'air-sewage' of the vilest quality, the imperfectly removed emanations from hundreds or thousands of human bodies." It is far from Dr. Ransome's intention, he says, to increase the scare about the infectiveness of phthisis. He urges that direct infection from patient to patient is of the rarest oocurrence in temperate climates. His crusade is against the retention of gross impurities in the air, hence his attack on the filthy habits of communities and "the vile conditions of our public assembly rooms."

### London Water Again.

IN the very complete and elaborate Annual Report of the Medical Officer of Health to the London County Council a matter is dealt with which is of considerable interest at the present time, namely, the safety of the water supply. Sir Edward Frankland having in his report stated that zymotic diseases traceable to the water supply had been absent in London since 1866, Mr. Shirley Murphy points out that the fact that pathogenic organisms were not found in the water at the intakes of the water companies, must not be accepted as a proof that these organisms were not from time to time present. All that can be assumed, he says, is that they were not present in sufficient numbers to be discovered in certain samples which, in relation to the volume of water at the intakes, were "infinitely small." Again, he draws attention to the statement made by Sir Edward Frankland to the effect that efficient sand filtration would prevent the passage of pathogenic germs into the filtered water, pointing out that, according to Sir Edward Frankland's own reports, the filters only very largely reduced the number of organisms and did not remove them altogether, and stating very properly that all depends on what is meant by the word "efficient." Clearly, if efficient filtration means the removal of all organisms, that is a thing which is as yet quite in the future. Further, as to the asserted absence of water-borne typhoid, Mr. Shirley Murphy says, "It is no doubt true that there has been no mortality from this cause sufficiently large to manifest itself in the absence of a proper system of observation; but, in view of the experience of 1894, I cannot assent to the statement as to 'the absence in London since the year 1866 of zymotic disease traceable to the water supply.'" What does Mr. Shirley Murphy mean, we wonder, by the words, "absence of a proper system of observation"? If we turn to the report of the water examiner, we shall, perhaps, find out. He says, in regard to the arrangements made by some of the companies, that "the filtered water collects in a general receptacle or well, and no means exist of determining the character of the filtration of each filter-bed," a plan the evils of which he insists upon; and it is well known that the authorities have no right to enter and see what is going on in the waterworks. But, we would ask, why should London be allowed to suffer from an "absence of a proper system of observation" in regard to such a matter as its water supply?

### Hospitals and Life Governors.

THE affairs of our hospitals are managed, as a rule, by boards and committees appointed out of the great mass of the governors. Broadly speaking, governors are of three kinds—annual subscribers, life governors who have given a considerable donation, and governors by virtue of some fund or other, sometimes a collection, sometimes a legacy, which they themselves have not contributed. We need hardly say that, for the sake of good and careful management, it is very undesirable that the governors whose interest is continuous, as shown by their continued annual subscriptions, should not be swamped by others whose appearance on the list of governors is more or less accidental, such as executors, presidents of football clubs, or lessees of theatres who have given a benefit for the hospital, or organisers of charity balls. A peculiarly flagrant attempt to "water" the list of life governors has just come to light at the Balmain Cottage Hospital in New South Wales. At the monthly meeting of the board a cheque for £86 12s. 1d. was paid as the result of a charitable ball given in aid of the hospital. Accompanying the cheque was a request that the names of eight persons mentioned who had taken an active part in arranging for the ball might be added to the hospital's list of life governors. After some discussion it was decided to take legal opinion before complying with the request. At the next meeting the president reported that he had obtained a copy of the opinion given by the late Attorney-General, which bore out the practice hitherto followed by the board in declining to recognise any such applications. He also cited a case which had been tested at Broken Hill some time ago, where workmen subscribing over £1 a year to the local institution in small periodical payments had claimed a vote. In that case also the Attorney-General had ruled that the donors were not entitled to the privilege of a vote. The result was that a resolution was carried to the effect that the persons mentioned be made life governors on the distinct understanding that this does not carry any voting privileges.

